# Service evaluation project: the effects of healthy weight commissioning for quality and innovation (CQUIN) interventions on metabolic parametrs of service users in a medium secure forensic ward

**DOI:** 10.1192/bjo.2021.846

**Published:** 2021-06-18

**Authors:** Elena Fage, Gaurav Sharma

**Affiliations:** 1 Brent Home Treatment Team; 2 St Bernards Hospital West London NHS Trust

## Abstract

**Aims:**

Service users of secure forensic units can be prone to weight gain due to various reasons including medications, physical illnesses, sedentary habits and mental health difficulties. They in turn are at greater risk of obesity related health problems like Diabetes, Hypercholesterolaemia, Ischaemic Heart Disease, Depression among others.

Our project was aligned with government's plan to improve prevention and screening for the obesity and metabolic syndrome among the patients of medium secure facilities by 2020.

Our primary objective was to gather and analyse the data on current metabolic parameters such as weight, body mass index (BMI), blood pressure (BP) and biochemistry markers of the service users on a 18 bedded Male medium secure long term rehabilitation ward.

Our secondary objective was to suggest healthy weight interventions that would help patients to loose weight and to explore the effects of these interventions on biochemistry markers and vital signs parameters.

**Method:**

We collected cross sectional data in given period of time (mid-March 2020). Seventeen service users were included in the final sample. Following initial data collection, we suggested various healthy weight interventions for the patients and repeated data collection after four months (July 2020).

Interventions offered:

Healthy eating group

1:1 sessions with doctors and pharmacists

Gym referrals

Dietician referrals

Relaxation group

**Result:**

Baseline

Fourteen patients on the ward (n = 14) were found to be either overweight or obese. Two patients (n = 2) had high BP, twelve patients (n = 12) had deranged lipid profile, six (n = 6) had high blood glucose (existing Diabetes).

Following intervention

Fourteen patients (n = 14) remained either overweight or obese. Nine patients (n = 9) lost weight following the intervention. Eight patients (n = 8) gained weight over 4 months. In both of the patients (n = 2) with raised BP the readings came back to normal after the intervention. One (n = 1) patient with normal BP at the baseline had high blood pressure following intervention.

**Conclusion:**

Our service evaluation projects revealed that majority of the patients on the ward had deranged metabolic parameters such as increased BMI, abnormal blood tests and high BP.

Following our intervention more than a half of the patients lost weight whilst other half gained weight during the period of observation, which we suspect is associated with significant physical activity restrictions during the coronavirus pandemic. In both patients with high BP at the baseline it has improved following the intervention.

We would continue implementing healthy weight interventions on the ward and across the unit.

